# Comprehensive Analysis of the Pectate Lyase Gene Family and the Role of *FaPL1* in Strawberry Softening

**DOI:** 10.3390/ijms241713217

**Published:** 2023-08-25

**Authors:** Yuanxiu Lin, Hao He, Yanling Wen, Shuaipeng Cao, Zisen Wang, Ziqing Sun, Yunting Zhang, Yan Wang, Wen He, Mengyao Li, Qing Chen, Yong Zhang, Ya Luo, Xiaorong Wang, Haoru Tang

**Affiliations:** College of Horticulture, Sichuan Agricultural University, Chengdu 611130, China; linyx@sicau.edu.cn (Y.L.);

**Keywords:** pectate lyase, strawberry, fruit softening, fruit firmness

## Abstract

Fruit softening is a crucial factor that controls shelf life and commercial value. Pectate lyase (PL) has a major role in strawberry fruit softening. However, the *PL* gene family in strawberry has not been comprehensively analyzed. In this study, 65 *FaPL* genes were identified in the octoploid strawberry genome. Subcellular localization prediction indicated that FaPLs are mostly localized to the extracellular and cytoplasmic spaces. Duplication event analysis suggested that *FaPL* gene family expansion is mainly driven by whole genome or segmental duplication. The *FaPL* family members were classified into six groups according to the phylogenetic analysis. Among them, *FaPL1*, *3*, *5*, *20*, 25, *42*, and *57* had gradually increased expressions during strawberry fruit development and ripening and higher expression levels in the fruits with less firmness than that in firmer fruit. This result suggested that these members are involved in strawberry softening. Furthermore, overexpression of *FaPL1* significantly reduced the fruit firmness, ascorbic acid (AsA), and malondialdehyde (MDA) content but obviously increased the anthocyanins, soluble proteins, and titratable acidity (TA), while it had no apparent effects on flavonoids, phenolics, and soluble sugar content. These findings provide basic information on the *FaPL* gene family for further functional research and indicate that *FaPL1* plays a vital role in strawberry fruit softening.

## 1. Introduction

Due to its rich nutrients (such as vitamin C, vitamin A, anthocyanins, etc.), unique flavor, sweetness, and bright color, the strawberry (*Fragaria × ananassa* Duch.) is cultivated and consumed worldwide. However, the extreme soft texture of strawberry brings a very short shelf life and poor fruit quality, thus leading to enormous marketing and economic losses. Therefore, the mechanisms underlying fruit softening and its manipulation are of great interest for both consumers and breeders.

Softening, a typical characteristic of ripening in most fleshy fruits, is a crucial factor that controls shelf life and commercial value. In certain species and cultivar, some degree of softening is desirable to consumers. However, during fruit ripening, excessive softening often leads to postharvest damage or infection decay that results in diminished fruit quality and significant economic losses. Although softening has been shown to be regulated by endogenous phytohormones (e.g., ethylene and abscisic acid) [1,2] and degradation of starch [3], it is mainly caused by modification or remodeling of the cell wall (CW) [4]. The plant CW comprises the primary CW, secondary CW, and middle lamella (ML). The primary CW consists of of cellulose, hemicellulose, and pectin; however, the weakening and disassembly of the CW are predominantly due to the solubilization and depolymerization of hemicellulose and pectin.

Pectin is the most complex polysaccharide and is also an important component of plant primary CW and ML. It plays an important role in intercellular adhesion, maintaining the stability, strength, and integrity of the CW. Accompanied with pectin degradation, the CW dissociates, and the fruit softens. The degradation of pectin is the result of the joint action of several metabolic enzymes, including pectin methylesterase (PME), polygalacturonase (PG), and pectate lyase (PL). PG is one of the most studied enzymes, which can hydrolyze homogalacturonan (HG), leading to the degradation of pectin. However, silencing of the ripening-related PG genes has a relatively small effect on slowing down the rate of fruit softening, and PG enzyme activity is relatively low in strawberries [5]. Hence, it is generally believed that the role of PG genes in strawberry fruit softening is relatively small. Recent studies have shown that there are a total of 82 PG genes in the strawberry genome of which *FaPG1* and *FaPG2* are highly expressed in the fruit. Silencing *FaPG1* and *FaPG2* significantly increases the firmness of strawberry fruit [6,7,8], indicating that these two genes play an important role in strawberry fruit softening. Similarly, PME has also been screened and identified in strawberry. In total, 54 PME genes have been identified in the strawberry genome of which *FvPME38* and *FvPME39* are involved in fruit softening and are regulated by MYB transcription factors [9,10]. However, the screening of the PL gene family in strawberry and the identification of its members related to fruit softening have not been reported yet.

Pectate lyase (PL) belongs to polysaccharide lyase family 1 (PL1), can randomly breaks the β-1,4 glycosidic bond, producing unsaturated C4-C5 bond oligomeric galactose uronic acid, thereby degrading pectin. PL exists in many plant species and has been identified in tomato [11], *Arabidopsis* [12], peach [13], and grape [14]. Its important role in fruit softening has also been confirmed in several species, such as banana [15], tomato [16], mango [17], and grape [18]. Previous studies have shown that silencing of the PL gene in tomatoes reduces the content of water-soluble pectin, which significantly improves fruit firmness and prolongs the storage period of the fruit [19]. In strawberry, it has been previously reported that the expression levels of three members of the PL family (*FaPLa*, *FaPLb*, and *FaPLc*) increased along with fruit maturation, indicating these genes are associated with fruit ripening and softening [20,21]. Meanwhile, by antisense inhibition of *FaPLc* gene expression, the fruit firmness was significantly increased [22,23], confirming the involvement of the PL gene in strawberry fruit softening.

Although a few softening-related PL genes in strawberry have been reported, the genome-wide systematic examination is still missing. In this study, we identified all the PL family members in strawberry; the basic information and expression profiles were obtained during fruit ripening. In addition, transient overexpression was used to clarify the function of *FaPL1* in strawberry fruit softening. The findings provide a foundation for further investigation of the function of PL family members in strawberry fruit softening, aimed to better reveal the molecular mechanism underlying strawberry fruit softening.

## 2. Results

### 2.1. Identification of PL Genes in Strawberry

**Figure 1:**
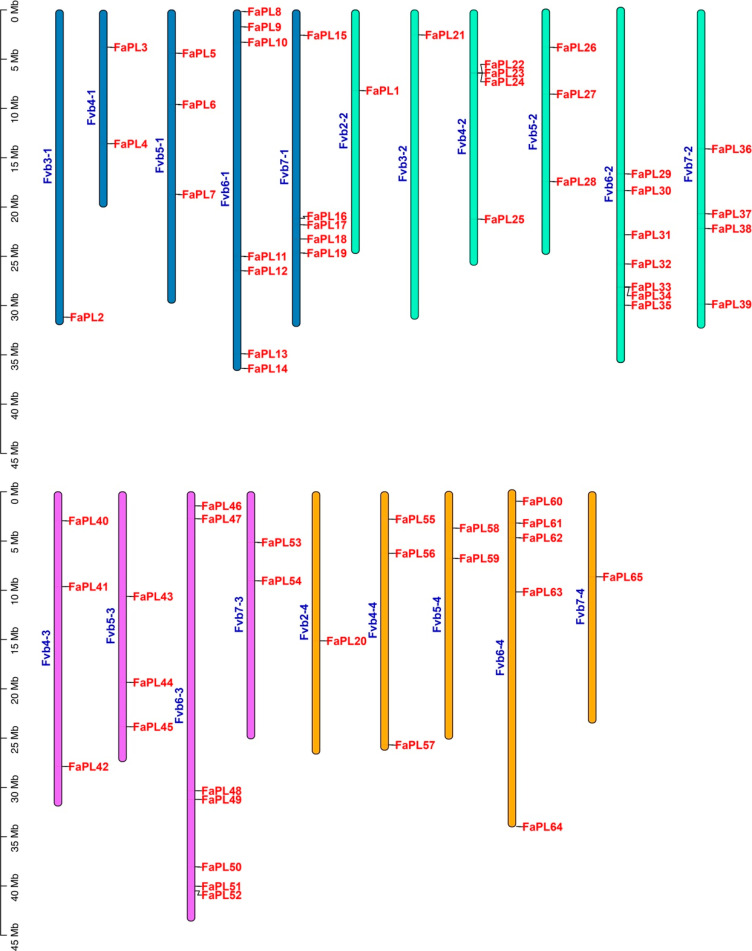
Chromosomal distribution and location of *FaPLs* in strawberry. Different colors indicate the chromosomes from different subgenomes of cultivated strawberry.

A total of 65 *FaPL* genes were identified by searching and confirming the conserved PL domains (PF00544) in the genome of cultivated strawberry. According to their distribution order on chromosomes, all the 65 *FaPL* genes were renamed as *FaPL1* to *FaPL65* (Figure 1). The sixty-five *FaPL* genes were unevenly distributed across the twenty-eight chromosomes in the four subgenomes of cultivated strawberry, with an apparent concentration on the chromosome 6. A maximum of seven *FaPL* genes were located on chromosome 6 from the first, second, and third subgenomes (Fvb6-1, Fvb6-2, Fvb6-3), while the minimum number was 1 on chromosomes Fvb3-1, Fvb2-2, Fvb3-2, Fvb2-4, and Fvb7-4. However, there were no *FaPL* genes on chromosomes 1 and 2 from the first subgenome (Fvb1-1, Fvb2-1), chromosome 2 from the second subgenome (Fvb2-2), chromosomes 1, 2, and 3 from the third genome (Fvb1-3, Fvb2-3 and Fvb3-3), and chromosomes 1 and 3 from the fourth subgenome (Fvb1-4 and Fvb3-4), which is not shown.

The characteristics and physicochemical properties of the deduced 65 FaPL proteins is shown in Supplementary Table S1. The number of amino acids varied from 91 to 500 aa, most of them (46) were concentrated from 400 to 500 aa. There were only two FaPL proteins comprising amino acids below 100 aa. The molecular weights (MW) were from 10.282 to 53.933 KDa. Only 21 FaPL proteins had isoelectric points (pI) below 7, while the others were all above or equal to 7 and three of which had pI above 10. Furthermore, based on the subcellular localization prediction results (Supplementary Table S1), most FaPL proteins were predicted to be located in the extracellular space (21), suggesting they might be secreted proteins. Several FaPL proteins were located in the cytoplasm (13), plasma membrane (9), and vacuolar (8); some FaPLs were localized in mitochondria, chloroplast, and peroxisome. Interestingly, a few FaPLs were predicted to be dual-localized, as examples, FaPL24 was located in both chloroplast and nuclear, while FaPL25 and FaPL57 were located in chloroplast or vacuolar (Supplementary Table S1). Subsequently, the origins of duplication events of *FaPLs* in strawberry were detected using MCScanX package. As a result, three types of duplication events were found, including whole genome duplication or segmental (WGD/segmental), dispersed, and proximal (Supplementary Table S1). Most *FaPLs* were duplicated by WGD/segmental, only seven and two *FaPLs* were duplicated from dispersed and proximal duplication events separately.

### 2.2. Phylogenetic and Gene Structure Analysis for FaPL Genes

**Figure 2:**
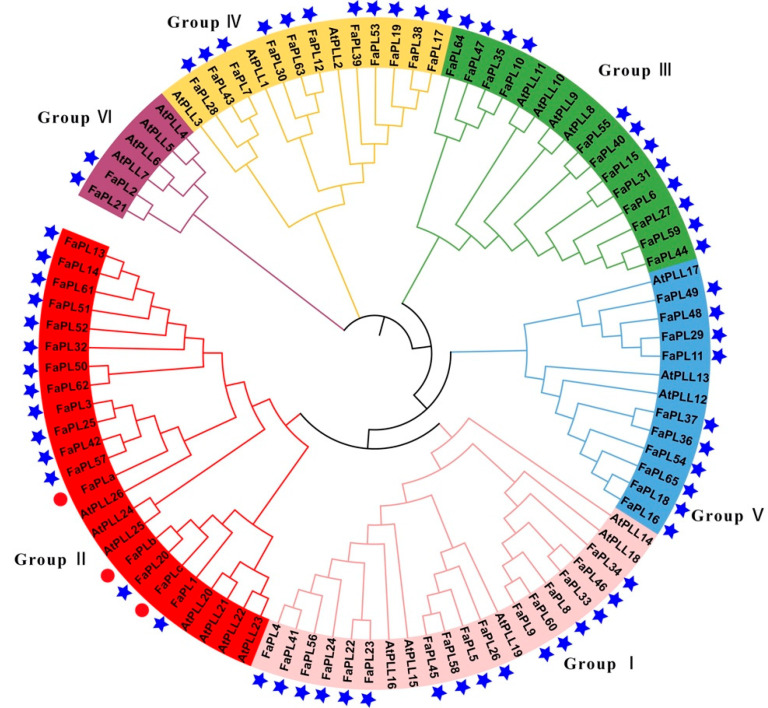
Phylogenetic tree of *FaPLs* from strawberry and *Arabidopsis*. Different branch colors represent the different groups. PL family members from strawberry identified in this study are marked with blue stars. The red dots indicate the previously reported *FaPLs*.

According to the result (Figure 2), all the sixty-five *FaPL* genes were classified into six clusters. Among them, group I is the largest group containing 16 members, followed by groups II and III, which had 14 and 12 members of *FaPL* genes, respectively. Both groups IV and V had ten *FaPL* members, whereas there were only two *FaPLs* included in group VI.

**Figure 3:**
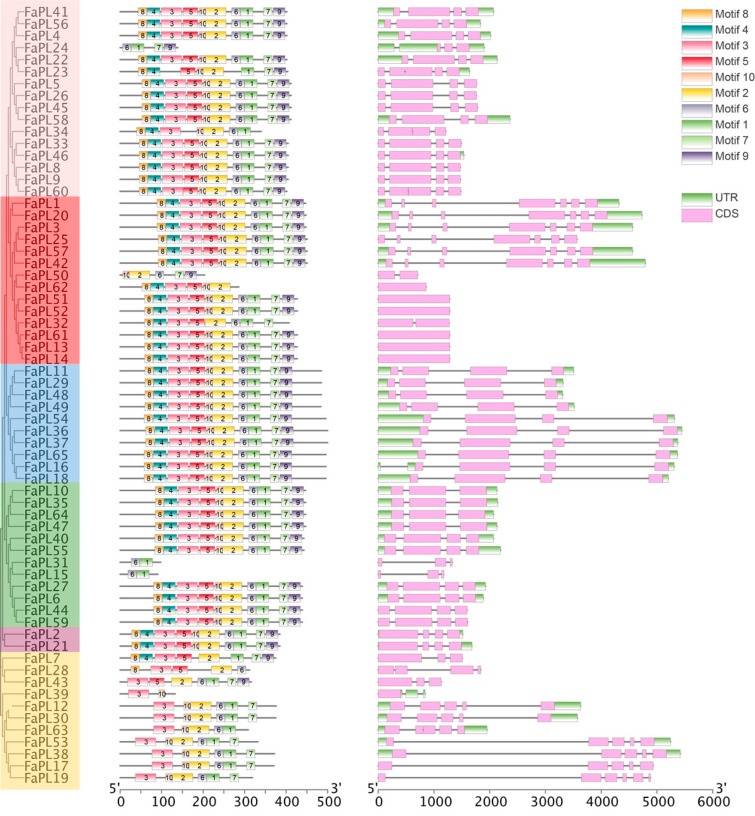
Conserved motifs and gene structure analysis of *FaPLs*. Left part indicated an unroot tree of strawberry *FaPLs*, middle part displayed the distribution of conserved motifs on each FaPL protein, and the right part showed the exon–intron distribution of *FaPLs*.

To better elucidate the structural characteristics of the *FaPL* genes, their intron/exon distributions were analyzed and visualized (Figure 3). Overall, six *FaPLs*, including in Group II, had no intron, and other members displayed discontinuous sequences due to the distribution of different number of introns. The exons numbers ranged from 1 to 7. Specifically, all members belonging to group V had four exons. It was noted that six *FaPLs* members (*FaPL1, 20, 3, 25, 57, and 42*) in Group II contained the most exons, while the other eight members of *FaPLs* only had one or two exons. The exons ranged from three to five of *FaPLs* divided into other groups. Meanwhile, the conserved motifs of FaPL proteins were analyzed using MEME Suite online software (version 5.5.3). The motifs number and distribution order were similar in FaPL members, most of which contained 10 motifs. However, FaPL15, 31, and 39 only contained two conserved motifs, FaPL50 had five motifs, and most members classed into group IV had six motifs (Figure 3). As shown (Figure 3), all the FaPL proteins contained the core motifs 1, 2, 6, or 10, which were annotated as PL domains. Motif 3 encoded a zinc finger domain, while the others were unknown.

### 2.3. Collinearity Analysis

**Figure 4:**
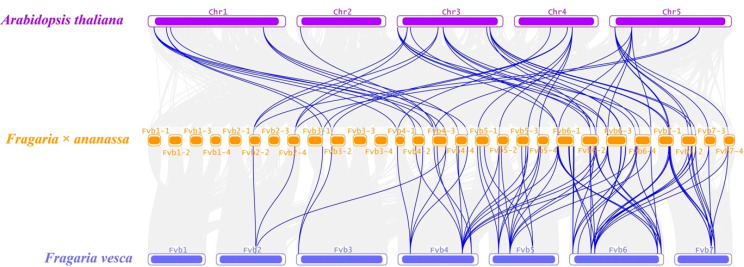
Collinearity analysis of *PL* genes among *Arabidopsis thaliana*, *Fragaria × ananassa*, and *Fragaria vesca* genomes. Grey lines indicate collinear blocks within the three genomes, while the blue lines represent collinear *PL* gene pairs. The purple, yellow, and blue columns indicate the chromosomes from *Arabidopsis thaliana*, *Fragaria × ananassa*, and *Fragaria vesca* genomes, respectively. Chromosome numbers are displayed at the side of chromosomes.

The collinearity analysis among *Arabidopsis*, woodland strawberry (*Fragaria vesca*), and cultivated strawberry (*Fragaria × ananassa*) was carried out to explore the evolutionary relationship of *FaPLs*. According to the result, *57 FaPLs*, 18 *AtPLs*, and 16 *FvPLs* were involved to form 129 collinear pairs (Supplementary Table S2). In particular, 52 pairs between *Arabidopsis* and cultivated strawberry and 57 between woodland strawberry and cultivated strawberry are highlighted in Figure 4.

### 2.4. Expression Profiles of FaPLs during Fruit Development and Ripening

**Figure 5:**
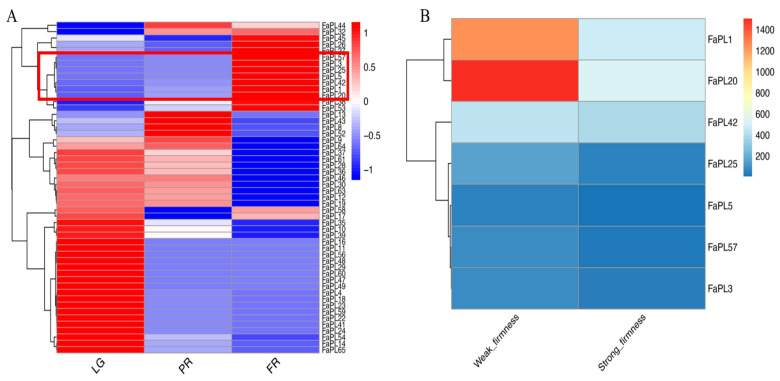
Heat maps showing the transcriptome-based expression of *FaPLs*. (**A**) The RNAseq retrieved expression during fruit development and ripening. A three-color scale was used with blue, white, and red indicating lowly, intermediately, and highly expressed genes, respectively. (**B**) Transcript abundance of *FaPL* genes in fruit with contrasting firmness. The color scale indicated the expression levels in FPKM value. LG, large green; PR, partial red; FR, full red. Red box indicates the seven *FaPL* members comprising gradual increases of expression during fruit development and ripening.

To identify the *FaPLs* related to strawberry fruit ripening, their expression patterns during fruit development were examined based on transcriptome data. As shown in Figure 5A, 52 out of 65 *FaPLs* distinctly expressed during the fruit development. Interestingly, most *FaPL* genes were highly expressed in the large green (LG) stage, while barely expressed in the partial red (PR) and full red (FR) stages. On the contrary, there were seven *FaPLs* (*FaPL1*, *FaPL3, FaPL5, FaPL20, FaPL25, FaPL42, FaPL57*) that exhibited lower expressions in the LG stage and gradually increased as the fruit ripened, indicating that they may be associated with strawberry fruit ripening. Subsequently, the expressions of these seven *FaPLs* were further assessed in fruit in comparison with firmness. As a result (Figure 5B), all of them had much higher expression levels in fruit with weak firmness than that in fruit with strong firmness, confirming their potential role in strawberry fruit softening. The transcriptome FPKM values are listed in Supplementary Table S3.

### 2.5. Functional Analysis of FaPL1 in Strawberry Fruit Softening

**Figure 6:**
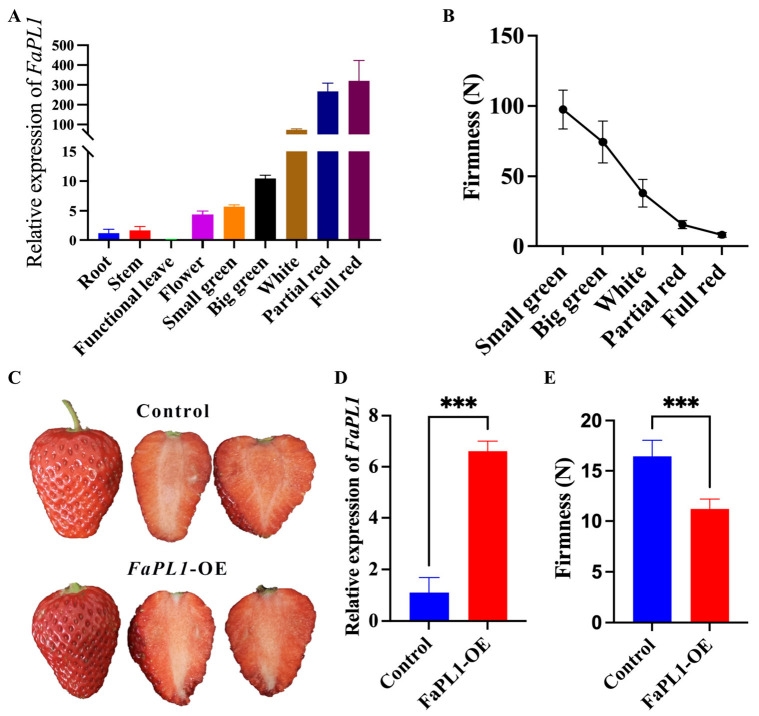
qRT-PCR based expression analysis and overexpression of *FaPL1* in strawberry. (**A**) The expression patterns of *FaPL1* in different tissues and during fruit development and ripening. (**B**) The change of fruit firmness of strawberry. (**C**) The phenotype of strawberry injected with empty (control) and *FaPL1*-overexpressing recombinant plasmid. (**D**) The relative expression of *FaPL1* in the full red *FaPL1*-overexpressed fruit and control fruit. (**E**) The firmness of full red *FaPL1*-overexpressed fruit and control fruit. OE, overexpressing. Triple asterisk indicated statistical difference at *p* ≤ 0.001 level.

Among all the seven potential softening related *FaPLs*, *FaPL3*, *FaPL25*, *FaPL42*, and *FaPL57* were close to the previously reported *FaPLa*, *FaPL20* was close to the previously reported *FaPLb* (Figure 2), while *FaPL5* had the lowest expression (Figure 5B and Table S3). Therefore, the *FaPL1* was selected for further expression and functional analysis. The temporal and spatial expression analysis result (Figure 6A) revealed that *FaPL1* expressed in various tissues, with the lowest level in functional leaves (the fully expanded leaf) and the highest level in fruit. In addition, the expression of *FaPL1* gradually increased along with fruit development and ripening, which showed a complete negative correlation with fruit firmness (Figure 6B). These findings suggested that the *FaPL1* may play a vital role in strawberry fruit softening. Furthermore, *FaPL1* was transiently overexpressed in strawberry fruit to validate its function in softening. The phenotypic result showed that overexpression of *FaPL1* did not apparently affect the fruit skin color (Figure 6C). qRT-PCR analysis of *FaPL1* expression indicated a seven times higher level in the overexpressed sample compared to the control (Figure 6D), suggesting *FaPL1* was successfully overexpressed. Moreover, the fruit firmness was significantly decreased by overexpression of *FaPL1* (Figure 6E), confirming the important role of *FaPL1* in strawberry softening.

### 2.6. The Effects of FaPL1 Overexpression on Fruit-Ripening-Related Traits

**Figure 7:**
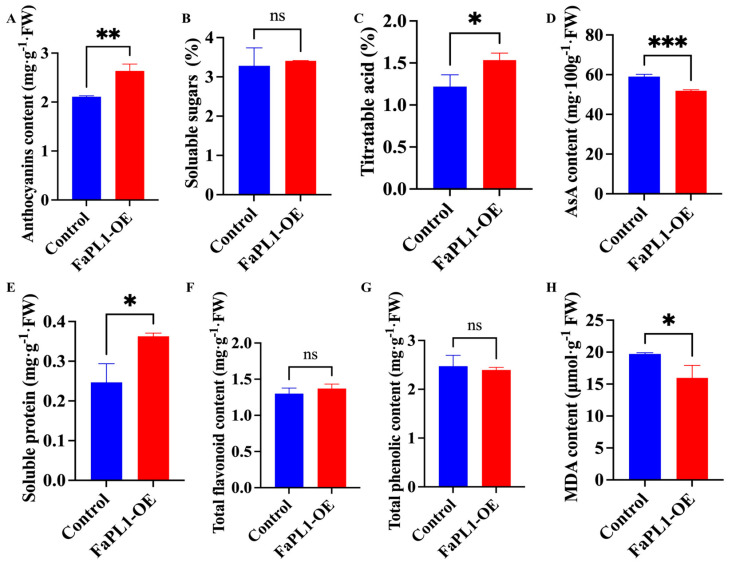
The effects of *FaPL1* overexpressing on the ripening-related traits. (**A**–**H**) indicate total anthocyanins content, soluble sugar, titratable acidity, AsA, soluble protein, total flavonoid, phenolic, and MDA content, respectively. Single, double, and triple asterisks indicate statistical differences at *p* ≤ 0.05, 0.01, and 0.001 levels. ns, no significant difference was found.

According to the result, the content of total anthocyanins, titratable acidity (TA), and soluble protein was remarkable higher in *FaPL1*-overexpressed fruit than that in the control fruit (Figure 7A,C,E). By contrast, AsA and malondialdeehyde (MDA) contents exhibited significantly lower levels in the *FaPL1*-overexpressed fruit compared to the control fruit (Figure 7D,H). However, the contents of soluble sugar, total flavonoid, and phenolic were similar in *FaPL1*-overexpressed fruit and the control fruit, which showed no obvious differences (Figure 7B,F,G).

## 3. Discussion

Due to their important roles in a broad range of physiological processes associated with pectin degradation, such as plant growth, development, fruit softening and ripening, PL genes have been identified in various plant species. It has been reported that a total of 26, 20, 12, 46, 22, and 16 *PL* family members were identified in *Arabidopsis* [24], peach [13], rice [25], *Brassica rapa* [26], tomato [19], and grape [14], respectively. In strawberry, several *FaPL* genes were obtained from multiple varieties, including *FaPLa*, *FaPLb*, and *FaPLc*, from ‘Chandler’, *FaSCPL* from ‘Sweet Charlie’, *FaTPL* from ‘Toyonoka’, and five subtype sequences from ‘Elsanta’ [20,21,27]. However, to our knowledge, the genome-wide analysis of this family remains limited. In this study, 65 *FaPL* genes were identified in strawberry based on a genome-wide investigation, which is more than the numbers in the above-mentioned species. It is possibly because the cultivated strawberry has undergone a whole genome duplication during the evolutionary process [28]. Segmental and tandem are two main duplication events driving the expansion of gene families [29]. For example, *PpePL5*, *6*, *7*, and *8* have been regarded as arising from tandem repeats [13], while the *GhPELs* in cotton seemed likely to be driven by segmental duplication [30]. Here, we have found that most (55 out of 65) *FaPL* genes were duplicated from segmental events (Table S1), which may contribute to the gene family expansion and their diverse structures and functions. These results indicated different expansion mechanisms of the *PL* gene family among different species. Moreover, the unrooted tree separated the *FaPL* genes into six different groups (Figure 3), which is different from the five groups from peach [13], tomato, [19] and cotton [30]. This may be caused by the larger number of *PL* genes in strawberry. Except for Group II, the *FaPL* genes involved in the same group have similar exon–intron structures. Whereas, in Group II, a multiplicity of exon numbers was found (Figure 3), suggesting their probable functional differentiation.

Fruit ripening is a complex process that involves substantive alterations in gene expression resulting in changes in flavor, aroma, and texture. Being one of the important cell wall modification genes, the *PL* expression has been found to be related to fruit ripening in various species. For instance, the PL gene is mainly expressed in ripe fruit but not the unripe fruit of banana [31], the expression of five peach *PpePL* genes, and three strawberry *FaPL* genes accumulated during fruit ripening [13,20,22]. Consistent with the previous studies, we found that there were seven *FaPL* genes (*FaPL1, 3, 5, 20, 25, 42, and 57*) that have gradually increased expression patterns during fruit development and ripening (Figure 5). The predominant and high expression of *FaPL1* in fruit during ripening was also confirmed by a qRT-PCR experiment (Figure 6A), revealing these genes are associated with strawberry ripening. Additionally, it has been well documented that *PL* genes play a central role in fruit softening [13,14,19]. *FaPLa*, *FaPLb*, and *FaPLc* have been suggested to participate in strawberry softening. Silencing *FaPLc* resulted in 30% firmer fruit than the control [22]. Here, according to the phylogenetic tree (Figure 2), it was found that among the seven ripening related *FaPLs, FaPL3, 25, 42*, and *57* were classified into the same clade with *FaPLa, FaPL20* was clustered with *FaPLb, and FaPL1* was close to *FaPLc*. This result demonstrated these genes may have similar functions in strawberry softening. Furthermore, we found that all of the seven ripening related *FaPL* genes apparently had higher expression levels in the fruit with weak firmness, compared to the fruit with strong firmness (Figure 5B). Transient overexpression of *FaPL1* significantly decreased the strawberry firmness (Figure 6E), confirming its key role in strawberry softening. Notably, *FaPL5* was clustered with *AtPLL19* in Group I, which is different from the other six ripening related *FaPLs* (Figure 2). *AtPLL19* was identified by its xylem-specific expression in *Arabidopsis* [32]. Combined with the fact that *FaPL5* had the lowest expression among the seven ripening related *FaPLs* (Figure 5B), it can be speculated that *FaPL5* may mainly function in xylem vascular development rather than fruit softening, which needs further research.

Developing methods without influencing the edible and appealing aspects of fruit, including color, aroma, or nutritional value, has currently become the major goal for controlling softening [4]. It has been suggested that the antisense expression of the *FaPLc* gene in strawberry did not affect the fruit color [22]; while overexpression of *VvPL15* in tomato accelerated the fruit ripening and coloring [14]. In the present study, we found that overexpression of *FaPL1* significantly increased anthocyanin content (Figure 7A). The possible explanation is that *FaPL1* may have a similar function with *VvPL15* in promoting fruit ripening and coloring. This may also explain the decrease of AsA in *FaPL1*-overexpressed fruit (Figure 7D). Because it has been suggested that the AsA decreased during strawberry storage and senescence [33], the facilitation of ripening by overexpression of *FaPL1* may lead to fruit senescence faster than the control and thus contained a lower AsA content. In addition, we have also found that *FaPL1* overexpression increased that soluble protein content in strawberry (Figure 7E). This is probably because the overexpression of *FaPL1* caused the degradation of the cell wall, resulting in the release of proteins. Moreover, it has been reported that acidic pH can cause cell wall loosening by inducing *PL* expression [34]; however, how the overexpression of the *PL* gene increases the content of titratable acidity (Figure 7C) is still to be studied in the future.

## 4. Materials and Methods

### 4.1. Identification and Comprehensive Analysis of FaPL Genes

The genome of cultivated strawberry was downloaded from the GDR (Genome Database for Rosaceae) (https://www.rosaceae.org, accessed on 23 August 2023) [35]. The specific Hidden Markov Model (HMM) file for the PL conserved domain (PF00544) was downloaded from the Pfam database (https://www.ebi.ac.uk/interpro/, accessed on 23 August 2023) [36] and used as a query to search the candidate *FaPL* genes by the HMMsearch program. The e value was set to 10^−5^, and the other parameters were set as default. The sequences with complete PL domain were further confirmed by searching the NCBI conserved domain database [37]. The deduced amino acid number, molecular weight (MW), and isoelectric point (pI) of putative proteins were obtained using a perl script. The chromosome locations of *FaPLs* were retrieved from the genome annotation file; the conserved motifs were analyzed using the MEME suite online program (version 5.5.3) and visualized together with the gene structure using TBtoos software (version 2.001). The subcellular localization prediction was performed by WOLF PSORT program (https://wolfpsort.hgc.jp, accessed on 23 August 2023).

### 4.2. Phylogenetic and Evolutionary Analysis of FaPL Genes in Strawberry

Based on the multiple alignment of FaPL proteins obtained by the MUSCLE program, a phylogenetic tree was constructed by MEGA X software (version 10.1.8) using the maximum likelihood method [38]. The beautification of the tree was subsequently carried out using the iTol online tool (https://itol.embl.de/about.cgi, accessed on 23 August 2023) [39]. Duplication events and the collinear gene pairs were determined using MCScanX software (http://chibba.pgml.uga.edu/mcscan2/, accessed on 23 August 2023). All the analysis was conducted using the default parameters of specific software according to the user instructions.

### 4.3. Expression Analysis

The RNAseq-based expression profiles of *FaPLs* in different fruit developmental stages and in strawberry fruits with contrasting firmness were retrieved from the previously published transcriptome data PRJNA838938 (https://www.ncbi.nlm.nih.gov/sra/?term=PRJNA838938, accessed on 23 August 2023) and PRJNA662854 (https://www.ncbi.nlm.nih.gov/sra/?term=PRJNA662854, accessed on 23 August 2023), respectively. The expression level was represented by the FPKM values. The heatmap was created using “pheatmap” package of R software (version 4.2.2) with a normalization in row.

RT-qPCR-based expression analysis were carried out using SYBR Green Premix Ex Taq^TM^ (Takara, Tokyo, Japan) on a CFX96 RT-qPCR system (Bio-Rad, Hercules, CA, USA). The total RNA was extracted from the plant sample using the improved cetyltrimethylammonium bromide (CTAB) method. The strawberry tissues and fruit at different developmental stages were collected in the previous study [40]. The first strand cDNA was synthesized following the operating manual of PrimeScriptTM RT reagent Kit with gDNA Eraser (Takara, Tokyo, Japan). The relative expression was calculated using the 2^−ΔΔCt^ method [41]. The 26-18S interspacer RNA sequence [42] was used as the internal reference. Expression data was reflected by mean± standard deviation (SD) of three independent biological replicates. Specific primers used for RT-qPCR were designed using the NCBI online tools. All the primer sequences are listed in Supplementary Table S4.

### 4.4. Transient Overexpression of FaPL1 Gene

The full length of *FaPL1* CDS was amplified using the primers *PL1*-TF and *PL1*-TR (Supplementary Table S4) according to the sequence retrieved from the strawberry genome and was substantially homologous recombined into a modified overexpression vector pCAMBIA1301 [42]. The recombinant plasmid was transformed into the strawberry cv. ‘Benihoppe’ fruit at the white stage using the previous agrobacterium-mediated transformation method [42]. The agrobacterium GV3101 strain was cultured at 28 °C until the OD_600_ reached 0.8. Each fruit was injected with 500 µL of bacterial solution and placed into a cultivation incubator. The fruit injected with empty vector was used as the control. At least 20 fruits were injected for overexpression and the control group separately. The injection fruit side was samples after 7 days for further measurement.

### 4.5. Determination of Fruit Firmness, Soluble Sugar and TA

Fruit firmness was determined two times on each injection part side of the fruit by a Texture Analyzer TA XT2i (Stable Micro systems, Godalming, Surrey, UK) with a 5 mm diameter cylinder needle and a penetration depth of 10 mm. Firmness was expressed as newton (N). Soluble sugar content was measured by the previously described colorimetric method [43]. Around 0.1 g of frozen stored fruit was completely extracted in 1 mL distilled water. After that, the 250 µL of the extract was diluted into 750 µL distilled water and 250 µL 2% (*w*/*v*) anthrone-ethyl acetate. The mixed solution was subsequently added to 2.5 mL concentrated sulfuric acid and put in a boiled water bath for 1 min. After cooling it down to room temperature, the absorbance of the extraction solution was recorded at 620 nm using a spectrophotometer, and the soluble sugars content was quantified by comparison to an external standard. The TA content was estimated by titrating the fruit extract against 0.1 N sodium hydroxide (NaOH) to the end point of pH 8.2 (faint pink) and represented as citric acid percentage.

### 4.6. MDA and Soluble Proteins

The MDA was assayed according to the formerly described procedure with slight modification [43]. Briefly, 0.5 g of frozen fruit sample was completely homogenized with 10% trichloroacetic acid. After a 10 min centrifugation at 4 °C, the clear solution was mixed with 0.67% 2-thiobarbituric acid. The mixture was then placed into a water bath at 100 °C for 10 min and immediately cooled on ice. The absorption values at 450 nm, 523 nm, and 600 nm were read separately. The results were represented as µmol per g FW.

The soluble protein content was measured according to the previous study. In brief, 0.5 g of fruit sample was homogenized in 5 mL of distilled water. The upper phase was collected and added with CBBG. After centrifugation, the absorbance of the mixture was tested at 595 nm. The content of soluble protein was quantified by a standard curve constructed using bovine serum albumin (BSA) protein.

### 4.7. Total Flavonoid, Phenolic, Anthocyanin, and AsA Content

Based on the previously described procedure [44], approximately 3 g of fruit were extracted in 5 mL of 80% acetone for 1 h at room temperature. After centrifugation for 10 min at 4500 rpm, the supernatant was collected for total flavonoids and phenolic content measurement. The photographic densities of 415 nm and 650 nm were read for the calculations of total flavonoids and phenolic content, respectively. The quercetin and gallic acid were used as external standards to construct the calibration curves separately. The total flavonoid content was presented as mg quercetin per kg of FW, and the total phenolic content was expressed as g gallic acid per kg of FW.

The determination of total anthocyanins was performed by pH differential method [45]. As previously demonstrated, the fruit sample was extracted in an acetic acid: water: acetone: methanol (1:2:4:4) solution. The mixture was incubated at room temperature for 30 min and then placed into a 40 °C water bath for 4 h. The clear extract was added with KCl (0.025 M, pH 1.0) and sodium acetate and then detected by recording the absorption values of 496 and 700 nm. The content of total anthocyanins was expressed as g pelargonidin 3-glucoside (Pg3G) per kg of FW.

AsA content was detected following the procedure described by Jiang et al. [43]. The content of AsA was calculated using the photographic density of the fruit extract at 534 nm and expressed as g AsA per kg of FW.

### 4.8. Statistical Analysis

All experiments were carried out in three independent biological replicates. Experimental data were expressed as mean values ± SD. The statistical differences were analyzed using Prism 9 software. The differences between the overexpression and control groups were determined using *t*-test. The results with *p* value below 0.05 were considered as statistically significantly different.

## 5. Conclusions

To summarize, 65 *FaPLs* gene family members were identified in strawberry and characterized. Among those, *FaPL1*, *3*, *5*, *20*, *25*, *42*, and *57* are likely to function in strawberry softening due to their increasing expression during fruit development and ripening and higher expression in weak firmness fruit. Transient overexpression of *FaPL1* significantly reduced the fruit firmness, confirming its role in strawberry softening. This work provides a basis for better understanding the function of the *FaPL* gene family in fruit ripening and softening.

## Data Availability

Not applicable.

## Supplementary Materials

The following supporting information can be downloaded at: https://www.mdpi.com/article/10.3390/ijms241713217/s1.
